# The role of protein S-acylation in vascular injury associated with metabolic disorders

**DOI:** 10.3389/fendo.2025.1643008

**Published:** 2025-09-03

**Authors:** Yayun Wang, Wenhui Zhu, Wenfan Wang, Jiayi Zhang, Dongsen Hu, Huanmeng Shao, Yingtong zhou, Shan Wang, Linhua Zhao

**Affiliations:** ^1^ Changchun University of Chinese Medicine, Changchun, Jilin, China; ^2^ Beijing University of Chinese Medicine, Beijing, China; ^3^ Binzhou Medical University, Yantai, Shandong, China; ^4^ National Center for Integrative Medicine, China Japan Friendship Hospital, Beijing, China; ^5^ The Affiliated Hospital to Changchun University of Chinese Medicine, Changchun, Jilin, China

**Keywords:** protein palmitoylation, palmitic acid, metabolic disorders, vascular injury, diabetes mellitus

## Abstract

Protein palmitoylation represents a prevalent form post-translational lipid modification across various organisms. This reversible and dynamic cellular process is significant in regulating the transcription and expression of downstream target genes, as well as in facilitating signal transduction. Consequently, it affects various cellular activities, including innate immunity, inflammation, glucose metabolism, lipid metabolism, and functions of the brain and heart. Vascular injury emerges as a critical target organ affected by complications associated with metabolic diseases, and the palmitoylation modifications are implicated in numerous pathological processes. This review offers an overview of current understanding on protein palmitoylation and palmitic acid, emphasizing the influence of the palmitoylation modification on cellular signal transduction in metabolic diseases and exploring its connection with metabolism-related conditions such as diabetic cardiopathy, diabetic nephropathy, and fatty liver diseases. Palmitoleic acid modification holds great promise for tackling challenges related to drug specificity, off-target effects, and delivery mechanisms in the exploration of targeted palmitoleic acid modification therapy *in vivo*. Moreover, methodological challenges in the joint analysis and mining of large databases, including gene databases, as well as the objective evaluation of studies on the bidirectional regulation of diseases, necessitate further investigation. These insights may provide novel insights for the development of clinical therapeutic strategies.

## 1 Introduction

The protein palmitoylation is a highly conserved post-translational modification and represents a prevalent lipid modification of proteins *in vivo* (1–3). According to the linkage mode, protein palmitoylation can be categorized into three distinct types, including the S-palmitoylation, N-palmitoylation, and O-palmitoylation (4, 5). S-palmitoylation involves the attachment of medium-chain or long-chain fatty acids to specific cytosolic cysteine residues within proteins. This modification is mediated by a family of S-acyltransferases that contain a conserved aspartate-histidine-histidine-cysteine motif (6–8).

**Figure 1:**
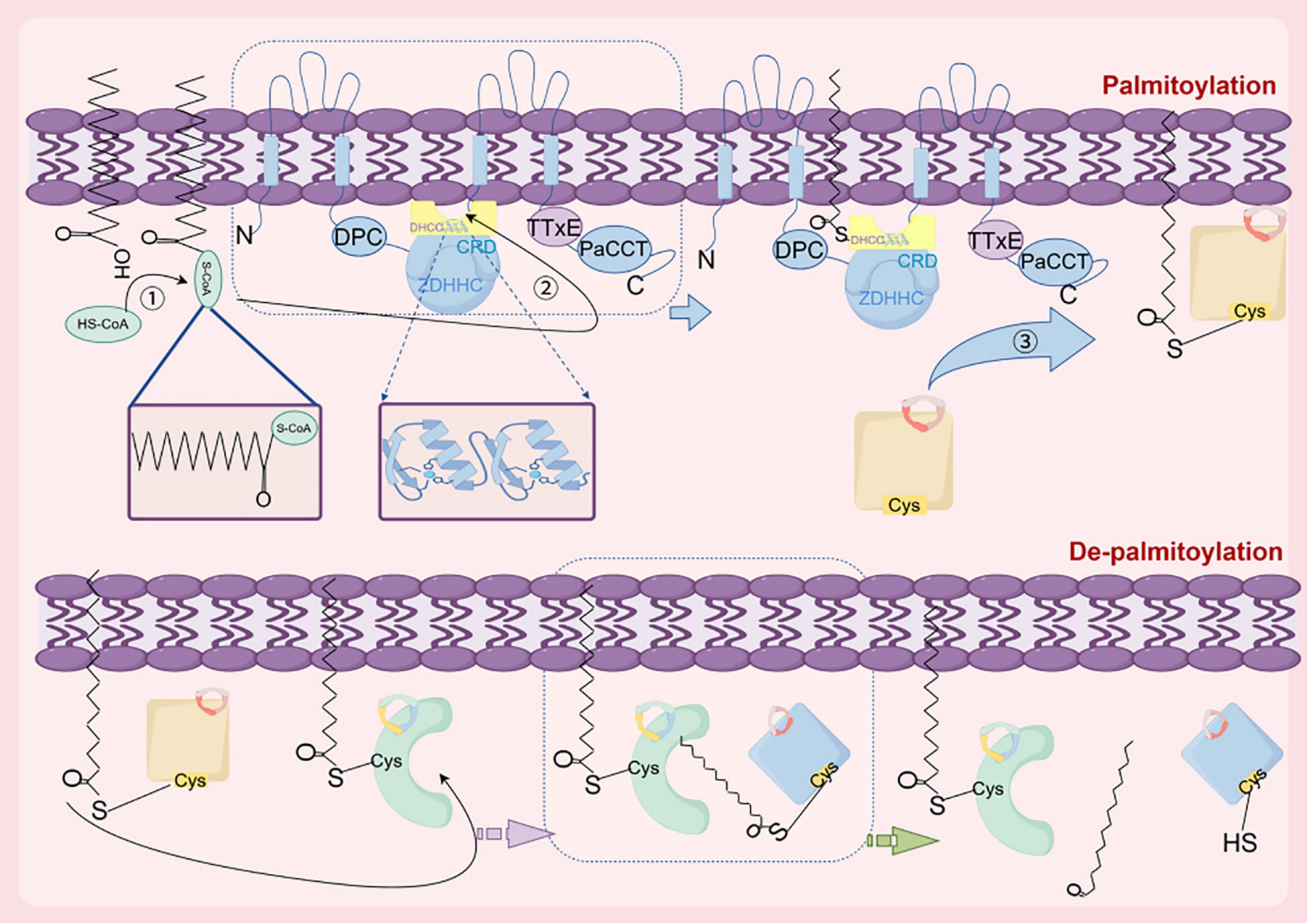
The primary components and processes involve in palmitoylation. Palmitoylation: initially, the DHHC cysteine within the active site undergoes self-palmitoylation by reacting with an acyl-coenzyme A donor to form an acyl-enzyme intermediate. Subsequently, acyl-coenzyme A is transferred to cysteine residues of target proteins, the ZDHHC protein is restored to its initial state, and an unstable thioester bond is formed by the protein substrate, thus achieve palmitoylation modification of the protein substrate. Depalmitoylation: APT1 contains a highly conserved serine-histidine-aspartate catalytic triplex structure and a hydrophobic pocket. The hydrophobic pocket is able to bind to palmitoylated modified proteins and localize the cysteine of the protein in the vicinity of the serine-histidine-aspartate catalytic triplex structure, thereby facilitating the depalmitoylation process and palmitate release from palmitoyl modified protein substrates.

Proteins palmitoylation and depalmitoylation can be rapidly cycled in an instantaneous manner, thus allowing rapid shuttling of proteins between specific organelles. Palmitoylation modifications are crucial for regulating various cellular processes, including protein stability, subcellular localization, membrane trafficking, interactions with effector proteins, and enzyme activity (9). Palmitoyl acyltransferases (PATs) are responsible for attaching palmitic acid to target proteins, and their catalytic reactions require palmitoyl-CoA as a substrate. Most PATs possess a cysteine-rich domain (CRD) consisting of 51 amino acids, which includes a highly conserved aspartate-histidine-histidine-cysteine (DHHC) catalytic structure (6). In mammals, the ZDHHC family consists of 23 proteins, named ZDHHC1-24 (excluding ZDHHC10). The ZDHHC proteins are mainly localized in membrane regions within the cell, such as the endoplasmic reticulum, Golgi apparatus, and endosomes, but a minority are also present in the plasma membrane. In addition, the process of the ZDHHC protein-mediated protein palmitoylation involves two critical steps. Initially, the ZDHHC undergoes autoacylation, wherein the cysteine residue in the DHHC-CRD domain covalently binds to palmitoyl coenzyme A, forming a palmitoyl enzyme intermediate. Although this palmitoylate intermediate can be hydrolyzed to release palmitic acid, the subsequent step of palmitoyl transfer is the more important. Specifically, the ZDHHC protein facilitates an enzymatic reaction, transferring its own bound palmitoyl group to the cysteine sulfhydryl group of the protein substrate. Concurrently, the ZDHHC protein reverts to its original state and the protein substrate forms an unstable thioester bond, resulting in the palmitoylation of the protein substrate (**Figure 1**) (4, 10).

On the other hand, the protein depalmitoylation refers to the enzymatic removal of palmitate thioester linkages from the cysteine residues of palmitoylated proteins. Depalmitoylating enzymes include acyl protein thioesterases (APTs), palmitoyl protein thioesterases (PPTs), and alpha/beta hydrolase structural domain 17 (ABHD17), which regulate the subcellular localization of proteins for plasma membrane or organelle transport and function (11, 12). Notably, APT1 and APT2 are two enzymes prominently associated with protein depalmitoylation. APT1 (LYPLA1) is a member of the highly conserved family of α/β hydrolytic enzyme family, and it is predominantly localized in mitochondria and exhibits significant depalmitoylation activity (13). APT1 is the earliest depalmitoylating enzyme found in Acyl Protein Thioesterases and widely expressed across many cell types. It regulates the depalmitoylation of G protein α-subunits, Ras-related proteins, and synaptic proteins, facilitating the hydrolysis of palmitoylthioester bonds from proteins to remove palmitic acid and making the palmitoylated modifications reversible. The modification process is reversible and maintains the dynamic balance of protein modifications, and the deficiency in this process can result in abnormal lipid metabolism, autophagy disorders, and neurodegenerative diseases (14). APT1 and APT2 not only catalyze the depalmitoylation of a large number of palmitoylation-modified proteins, but also regulate the dynamic balance between palmitoylation and depalmitoylation modifications by modifying cysteines to ensure their correct membrane localization and function, and participate in the transport process of peripheral membrane proteins. In addition, they utilize their hydrophobic pockets they contain to bind to proteins modified by palmitoylation and pinpoint the cysteines of these proteins in the vicinity of the serine-histidine-aspartate catalytic triad structure, thus facilitating depalmitoylation modification of palmitoylation-modified protein substrates and palmitate release (15).

Palmitic acid (PA), the most common saturated fatty acid in living organisms, is the energy source or component of some biochemicals and cellular structures (16).It is the first fatty acid produced during fatty acid synthesis and is a precursor to longer fat acids, which can also be converted to palmitic acid by excess carbohydrates in the body (17). Palmitoylation is the post-translational modification mode in which palmitic acid (C16:0) is covalently attached to cysteine residues of proteins via thioester bonds. Palmitate metabolism and protein palmitoylation are closely related biological processes, with the former providing a key acyl donor (palmitoyl-CoA) for the latter, which is involved in cell signaling, metabolic regulation, and other important physiological activities by modifying protein function. When palmitic acid synthesis is active, more palmitoyl-CoA may be generated, promoting protein palmitoylation; conversely, when catabolism is high, palmitoyl-CoA may be reduced, affecting the modification process (18).

**Figure 2:**
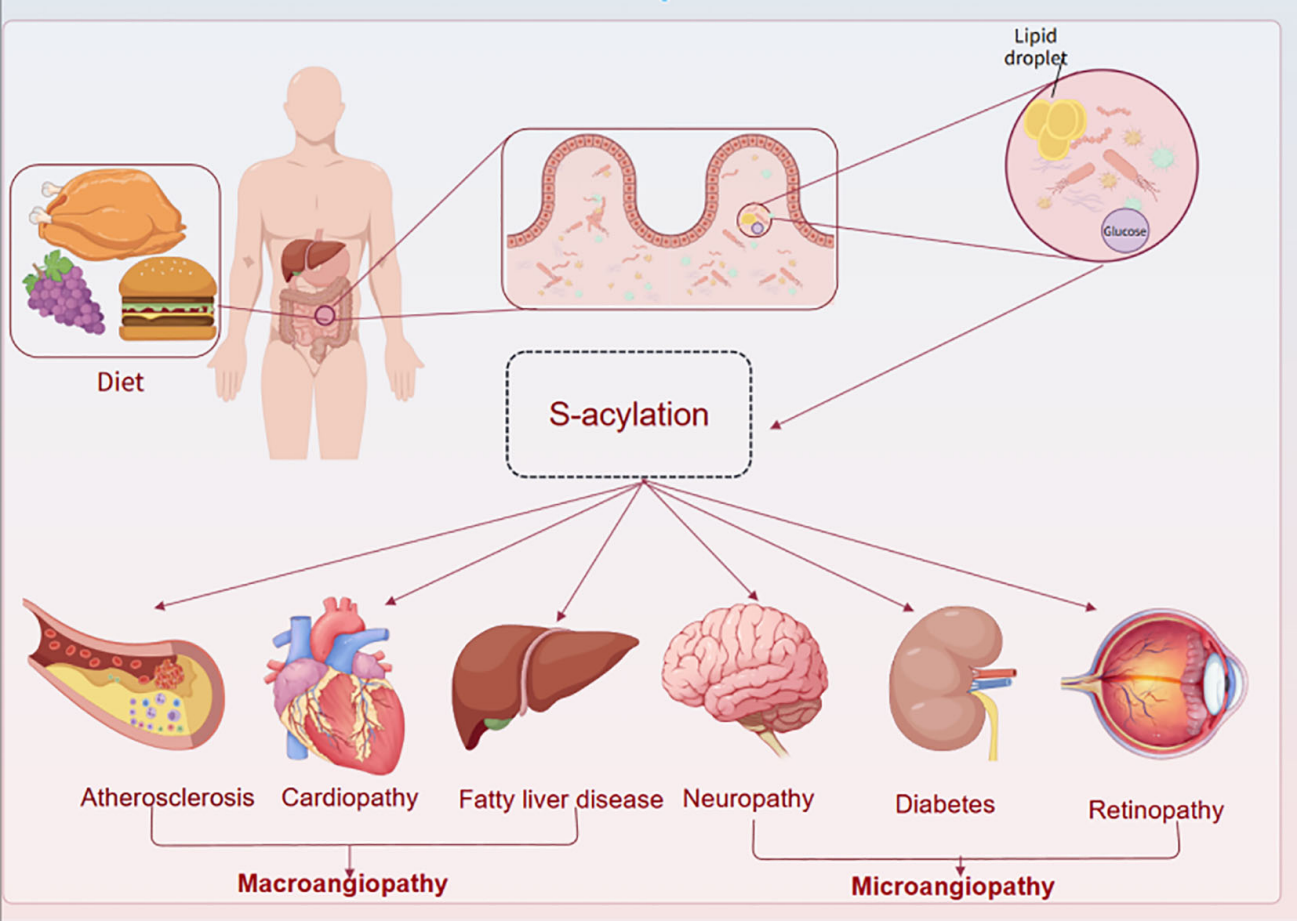
The whole vascular lesions of metabolic diseases involve the heart, brain, kidney, eye and peripheral system. There are different mechanisms in the large and microvascular disease systems in different organs, which affect vascular function.

Common metabolic diseases include type 2 Diabetes Mellitus (T2DM), obesity, non-alcoholic fatty liver disease (NAFLD), hyperlipidemia, as well as complications such as diabetic nephropathy, diabetic cardiomyopathy, their causes are related to many factors such as genetics, diet, exercise, aging and environment. Diabetic nephropathy and diabetic cardiomyopathy, whose etiology is related to many factors such as genetics, diet, exercise, aging, and the environment, and which can be slow-onset and have a long duration of treatment, have become the major chronic diseases around the world, causing an increasing number of public health problems. Epidemiology has found that more than 90% of diabetic patients have type 2 diabetes (19), in which vascular lesions are classified into macrovascular and microvascular lesions (20). Diabetic macrovascular lesions are common in coronary heart disease, stroke, and peripheral arterial disease due to atherosclerosis (21); microvascular lesions are common in diabetic nephropathy, diabetic retinopathy, and diabetic neuropathy (**Figure 2**) (22). The vasculature is the main damaged target organ in the pathological damage of many metabolic diseases. Due to the differences in hemodynamics, vascular structure, and diseased target organs, the pathological manifestations of the lesions show different degrees of vascular endothelial damage, vascular basement membrane thickening, microthrombosis, platelet and erythrocyte adhesion aggregation, and microcirculation disorders. In addition, differences in the energy metabolic state of different target organs, as well as differences in organ-specific growth factors or cytokines, are also important factors contributing to damage in these organs. Patients with metabolic diseases are chronically hyperglycaemic with insulin resistance, glucolipid metabolism disorders, inflammatory responses, and oxidative stress, which together lead to damage to the vascular endothelium and ultimately to vascular endothelial dysfunction (23). These factors disrupt the function and structure of the vasculature of the specific process is more complex, and the modification of proteins related to glycolipid metabolism, inflammation, and oxidative stress, of which palmitoylated due to the energy metabolism of the main involved in the adjustment of the part with a variety of biological regulatory properties can be a variety of forms of participation in the regulation of different pathologies.

Considering that S-acylation impacts the ability of proteins to interact at membrane interfaces, it is unsurprising that this post-translational modification affects numerous cellular processes, such as the functions of endothelial and cardiac cells, as well as cellular adhesion, growth, and division. The activity of adhesion molecules (24, 25), claudins, and desmosomal proteins 75 is contingent upon S-acylation. Palmitoylation regulates the cytoskeleton, cell proliferation and migration within smooth muscle cells. Palmitic acid may promote the proliferation of vascular smooth muscle cells and thus trigger atherosclerosis by altering palmitoylation modifications of relevant signaling molecules. Specifically, palmitic acid increases the expression of adhesion molecules such as VCAM-1 and ICAM-1 and inhibits endothelial-type nitric oxide synthase (eNOS) (26) activity through signaling pathways like TLR4/NF-κB, leading to reduced eNOS phosphorylation and NO bioavailability, thereby affecting vascular function. In addition, palmitic acid promotes platelet activation, increases thromboxane A_2_ (TXA_2_) synthesis (27), and decreases prostaglandin I_2_ (PGI_2_) levels, resulting in a procoagulant state, which in turn affects vascular structure and function. Junctional adhesion molecule C (JAM-C) is an immunoglobulin superfamily protein expressed in epithelial cells, endothelial cells, and leukocytes and is closely associated with leukocyte transendothelial migration, angiogenesis, and cell adhesion. Studies have shown that S-palmitoylation of JAM-C may be a potential target for controlling cancer metastasis (28).

Considering the distinctive characteristics of protein palmitoylation and its extensive biological functions, we primarily targeted in this review on vascular injury within metabolism-related diseases. We have thoroughly investigated the binding and interaction mechanisms between pathological injuries, including insulin resistance, oxidative stress, lipid metabolism abnormalities, and inflammation and various modification sites. Furthermore, we have referenced the characterization of various clinical drugs pertinent to palmitoylation modification. We hope this review would present a comprehensive overview of current research progress, aiming to provide valuable references for subsequent broader experimental investigation and clinical applications.

## 2 Palmitoylation modifies the type and course of vascular injury

### 2.1 Insulin resistance

Abnormal insulin action is a key factor in common diseases such as type 2 diabetes, obesity and insulin resistance (29). Palmitoylation is associated with cytotoxicity, can be reversed by APT1, and is associated with hypersecretion of insulin as well as beta-cell failure (30). Its damage to the vasculature is mainly characterized by glomerular basement membrane thickening and retinal capillary leakage in microvascular lesions, and atherosclerotic plaque formation and vascular calcification in macrovascular lesions. Abnormal levels of vasoactive substances such as ET-1 (31) due to decreased nitric oxide (NO) bioavailability, which in turn induces endoplasmic reticulum stress and mitochondrial dysfunction, leading to apoptosis of endothelial cells and ultimately endothelial dysfunction. In addition, the activation of oxidative stress leads to an increase reactive oxygen species (ROS) production and a massive depletion of antioxidant substances such as SOD (32), which puts the organism in a chronic low-grade inflammatory state. This can contribute to the release of excessive inflammatory factors, such as from adipose tissue, or lead to immune cell infiltration. Abnormalities in lipid metabolism are manifested by increased lipolysis and elevated levels of free fatty acids. Altered hemodynamics impairs endothelium-dependent vasodilatory function; microvascular dysfunction affects tissue perfusion and oxygen supply. At the same time, fibrinogen, coagulation factors and platelet activity are increased (33), promoting thrombosis; tissue-type plasminogen activator (tPA) activity is inhibited, and fibrinolytic function is diminished.

In the diabetic state, excessive accumulation of palmitate interferes with beta-cell function. The relationship between palmitate and insulin secretion has been demonstrated *in vivo* and *in vitro*, showing that insufficient insulin secretion leads to abnormalities in the insulin signaling pathway (34, 35). Palmitic acid induces pancreatic beta-cell dysfunction, which in turn triggers insulin resistance and diabetes mellitus (36–38). Due to diminished insulin action, fatty acid oxidation processes may be inhibited, leading to fatty acid accumulation and metabolic disorders. This would further exacerbate insulin resistance or increase oxidative stress and promote the development of metabolic diseases. In the context of insulin resistance, lipolysis of adipose tissue is enhanced, leading to elevated circulating levels of free fatty acids (FFA) (39). The elevation of FFA in insulin resistance is due to combined resistance to insulin-mediated inhibition of adipose tissue lipolysis and decreased adipocyte capacity for fatty acid capture in insulin-resistant states (40). Palmitoylation facilitates the translocation of endothelial eNOS from the cytoplasm to the mitochondrial membrane, This process enhances its activity and stabilizes its structure, ultimately increasing the production of NO (41). The regulation of protein palmitoylation by insulin affects endothelial cell function, while chemical inhibition of palmitoylation impedes insulin-induced angiogenesis *in vitro* (42). The hyperglycaemia induced by abnormal insulin function inhibits the activity of the palmitoylating enzyme DHHC-7, that leading to a reduction in palmitoylation, which in turn reduces NO secretion. This condition triggers endothelial dysfunction and vasoconstriction. Consequently, the palmitoylation of endothelial nitric oxide synthase is essential for the stimulation of nitric oxide release (43, 44). Besides, the conjunction of APT1 deficiency with hyperglycaemia lead to an increased palmitoylation. The APT1 activity is inhibited in the high-glucose environment, which coincides with the phenomenon of fibronectin accumulation in the vasculature. This situation impairs the process of deglutitional acylation in endothelial cells, which in turn triggers the phenomenon of vascular immaturity associated with defects in the function of proteins such as R-Ras (45).

### 2.2 Lipid metabolism abnormalities

A crucial pathway in energy metabolism is *de novo* liposynthesis, the process of synthesizing fatty acids from monosaccharides. This process is dependent on the catalyzing action of fatty acid synthase (FAS).Disorders of glucolipid metabolism impair the antilipolytic effect of adipose tissue on insulin, leading to increased lipolysis and increased release of free fatty acids (39, 46). In a state of insulin resistance, there is an increased FFAs flux to the liver, which stimulates the synthesis of very low-density lipoprotein (VLDL) particles, which in turn leads to elevated plasma levels of triglycerides (TG) and apolipoprotein B (Apo B) (47). Oxidative stress in the vascular wall causes oxidative modification of low-density lipoproteins (LDL), producing oxidized low-density lipoproteins (ox-LDL). At the same time, macrophages take up excess ox-LDL to form foam cells, while reduced levels of high-density lipoprotein (HDL) impair their anti-inflammatory and antioxidant functions to remove cholesterol efficiently, for example, in diabetic patients with combined atherosclerosis (48, 49). Abnormalities in lipid metabolism increase the activity of fibrinogen, coagulation factors, and platelets, which not only promotes thrombosis but also inhibits the activity of tissue-type fibrinogen activator. Although it is not clear how various modifications such as lipids alter fibronectin metabolism, leading to vascular instability, it has been shown that lipid modifications of proteins are associated with diseases such as infections, premature aging, cancer, and diabetes. Lipid modifications cover a variety of forms of fatty acylation, including n-myristylation, n-acylation, and s-acylation. Recent studies suggest an unexpected role for *de novo* lipogenesis in the S-palmitoylation of eNOS within blood vessels and the foam cells and inflammatory macrophages are critical contributors to the pathogenesis in metabolic disorders. The activity of the CD36-FABP4-p38-PPARδ signaling axis can be effectively attenuated by intervention with palmitic acid and its target, acyl-CoA synthase-1 (ACSL1). It offers a potential therapeutic strategy for preventing acute high-fat feeding (AHFF) induced macrophage foaming and inflammatory responses (50). The excess saturated fatty acids, such as palmitic acid, could trigger hepatic lipotoxicity and lead to vasculopathy in NAFLD, a process in which adipocyte apoptosis is regulated by multiple signaling pathways (51).

### 2.3 Oxidative stress

Abnormal metabolism leads the body to produce large amounts of ROS, and although a moderate increase in ROS is essential for signal transduction, overproduction triggers oxidative stress, which in turn leads to abnormal proliferation and migration of vascular endothelial cells and vascular dysfunction (52, 53). Injuries such as high glucose and high fat induce ROS production mainly through several pathways: activation of protein kinase C isozymes, increased formation of glycosylation end products (AGEs), and increased glucose flux through the aldose reductase pathway or the polyol pathway (54, 55). Hyperglycaemia induces binding of AGEs to receptors (RAGE) (56, 57), which activates NADPH oxidase, catalyzing the generation of superoxide from oxygen, and aldose reductase, which depletes NADPH, weakening antioxidant defenses via the polyol pathway (58). In addition, metabolic disorders deplete antioxidants such as glutathione (GSH), reducing the body’s antioxidant capacity and leading to a decrease in the activity of antioxidant enzymes such as SOD (59) and catalase (CAT) (60).

As palmitic acid leads to a significant increase in mitochondrial ROS production accompanied by mitochondrial DNA damage and dysfunction, apoptosis, and inhibition of insulin signaling, PA damage to mitochondria can be mitigated by inhibition of the mitochondrial autophagy-ROS-CTSB-NLRP3 pathway, which reduces lysosomal membrane permeabilization (LMP) and inhibits inflammation and cellular pyroptosis (61). In non-alcoholic steatohepatitis (NASH), the overall peroxiredoxin activity of peroxiredoxin reductase (PRDX) in the liver is significantly decreased, which is further exacerbated by palmitic acid (PA) by directly binding to PRDX1 and inhibiting its peroxidase activity (62). It was shown that ROS/JUN is a common response pathway for insulin resistance induced by fatty acids in HepG2 cells (63). Increased oxidative stress may exacerbate vascular injury by inhibiting the normal function of the antioxidant enzyme system through palmitoylation modifications. Palmitic acid activates NADPH oxidase, which in turn generates superoxide anion (O^_2_-^) and the lipid peroxide malondialdehyde (MDA) (64), which are end-products of palmitic acid oxidation, and can reflect the extent of vascular damage caused by lipid peroxidation. In addition, palmitic acid induces apoptosis in endothelial cells by activating endoplasmic reticulum stress and mitochondrial pathways. Excess palmitic acid may also increase intracellular oxidative stress by interfering with autophagic mechanisms, leading to further exacerbation of inflammatory responses (65). Elevated levels of palmitoylcarnitine suggest that mitochondrial β-oxidation is impaired, and thus increased oxidative stress may exacerbate vascular injury by inhibiting the normal function of the antioxidant enzyme system through palmitoylation modifications.

### 2.4 Inflammatory

Inflammation plays a key role in vascular injury in metabolic diseases and manifests itself in a variety of forms, with chronic inflammation and immune response being the most prevalent (66). This inflammation typically presents as an infiltration of immune cells, such as monocytes, macrophages, and T cells, and damage to the vessel wall. Damage to the vascular endothelium results in the release of additional inflammatory mediators, such as IL-8, MCP-1 (67), and NLRP3 (68), which attract monocytes and macrophages for further infiltration. The infiltrating cells transform into foam cells after phagocytosis of oxidized low-density lipoprotein (oxLDL) and release pro-inflammatory factors such as TNF-α, which in turn exacerbate insulin resistance and vascular sclerosis. MD2 has been shown to drive the inflammatory response in studies of inflammatory response and myocardial injury induced by factors such as high fat and high glucose (69). In addition, immune responses induced by intestinal flora, especially those triggered by short-chain fatty acids (SCFA) produced by the flora, play an important role in the pathogenesis of metabolic diseases such as diabetes (66). In addition, intestinal bacteria are able to convert carbohydrates and polysaccharides that cannot be broken down by the host itself into short-chain fatty acids (SCFA), a process that has been identified as an important potential metabolic target for glucose metabolism, insulin resistance, obesity prevention, and T2DM (70).

Vascular cell adhesion molecules (VCAM-1/ICAM-1) are key target proteins for palmitoylation regulation. In the presence of DHHC-15, the expression of these molecules on the surface of vascular endothelial cells is enhanced, thereby promoting leukocyte adhesion. Thus, excess palmitoylation accelerates atherosclerotic plaque formation. In addition, palmitoylation modifications may alter the function of tight junction proteins such as Zonula Occludens-1 (ZO-1) in endothelial cells, leading to an increase in vascular permeability and facilitating the infiltration of inflammatory factors, which in turn exacerbates vascular injury (71). It has been shown that palmitoylated CD36 receptors recognize ox-LDL (72, 73) and promote its uptake, thereby exacerbating the inflammatory response of the vascular endothelium. In studies of vascular smooth muscle cells, found that the zDHHC4 enzyme, when modified by palmitoylation, becomes localized on the surface of the cell membrane and binds directly to vascular endothelial growth factor (VEGF) and platelet-derived growth factor (PDGF). This binding inhibits the activation of these growth factor receptors, thereby blocking the formation of abnormal pathological neovascularisation (74). In addition, palmitoylation of Rab3 GTPase-activating protein 1 (Rab3gap1) by inhibiting zDHHC family activity or blocking it modulates the exocytotic release of neuropeptides and hormones from neuroendocrine cells, as well as secretion of atrial natriuretic peptide (ANP) from cardiac myocytes, resulting in an improvement of vasodilatory function in patients with heart failure (75, 76). Palmitoylation also promotes the formation of integrin adhesion plaques (24, 77), enhances smooth muscle cell migration to the vessel wall, and is involved in the process of development of multiple vascular injuries. Also, the effect of palmitoylation modification on L-type calcium channels alters their voltage sensitivity, and palmitic acid inhibits their activity, leading to decreased vascular contractility (78). Involvement of palmitoylation in the vascular epithelium The vascular epithelium is the outermost layer of the vascular wall and consists mainly of connective tissue, fibroblasts, adipocytes, nerve endings, and microvessles (79). Abnormal deposition and fibrosis of the extracellular matrix (ECM) causes the vessel wall to become stiff, which affects the diastolic function of the vessel. Matrix metalloproteinases (MMPs) play a key role in this process (80). It has been shown that palmitoylated MMP-2/9 with enhanced activity is able to degrade the vascular basement membrane, which in turn promotes plaque rupture. In addition, palmitoylation regulates diabetic retinopathy in db/db mice through activation of the NLRP3/NF-κB signaling pathway (81). This process promotes nuclear translocation followed by upregulation of IL-6 and TNF-α expression, exacerbating vascular inflammation, which may be a potential mechanism of atherosclerosis (82, 82). In addition, LPA is a bioactive lipid mediator that triggers inflammation through its receptors 1-6, further exacerbating vascular injury and fibrosis (83).

## 3 Exploration of targeted palmitoylation modifications in clinical therapeutics

The focus of clinical intervention strategies and research revolves around a deeper understanding of the characteristics of vascular injury in metabolic diseases (84) and an emphasis on the role of key mechanisms of clinical glucose and lipid-lowering therapy (85, 86). Endothelial dysfunction in patients is strongly associated with the outcome of vascular injury (87). Aggressive control of primary disorders of glucose-lipid metabolism, combined with early comprehensive vascular intervention, is the key to prevention and treatment. In addition to the widely recommended metformin, glucagon-like peptide-1 receptor agonists, and sodium-glucose cotransporter protein-2 inhibitors, research targeting the latest molecular mechanisms, such as aldose reductase inhibitors, peroxisome proliferator-activated receptor-gamma agonists, glucokinase agonists, and mitochondrial energy modulators, is also being actively pursued.

**Figure 3:**
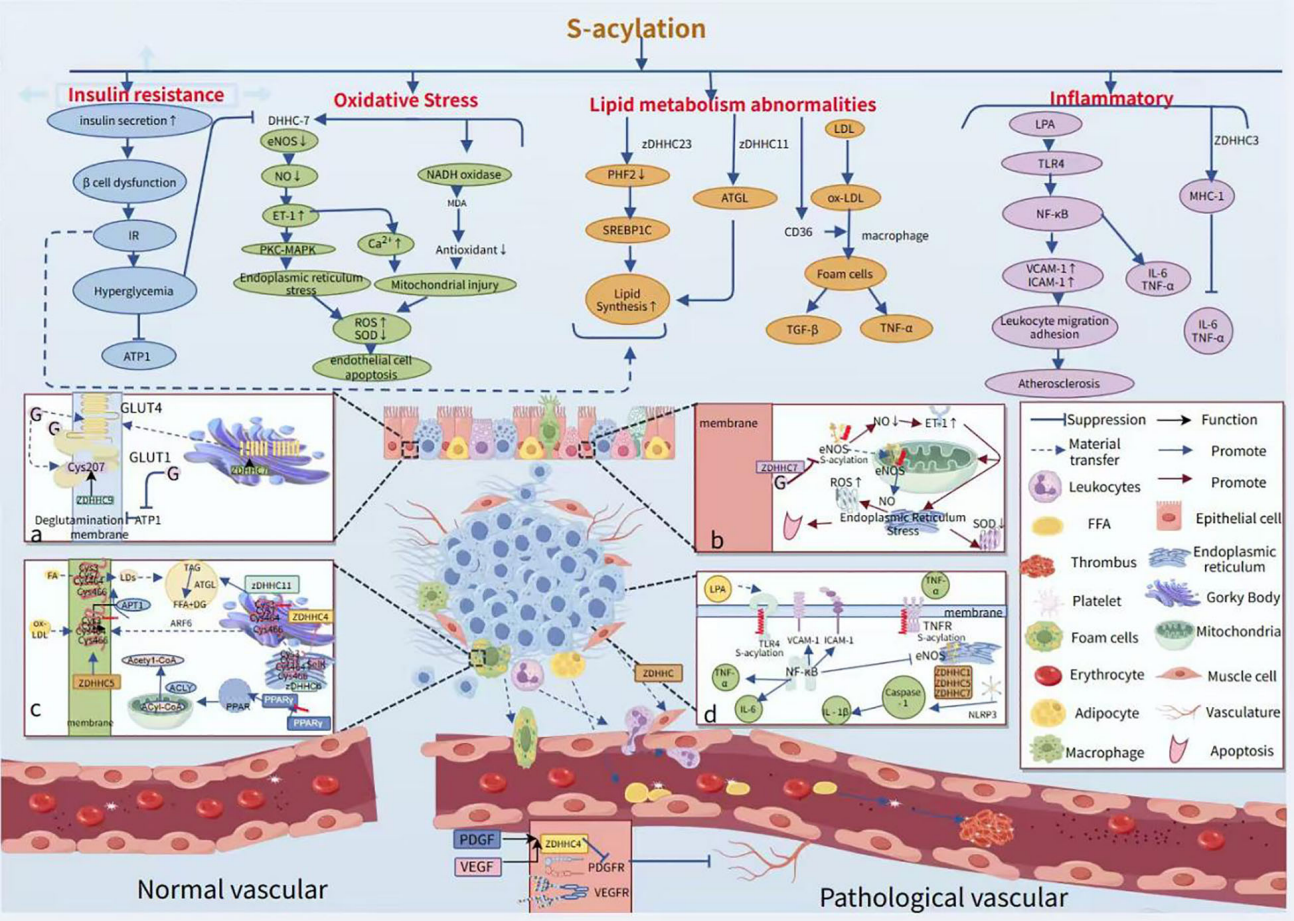
Role of palmitoylation in modifying vascular injury pathological phenotypes and processes. APTI deficiency results in peripalmitoylation, which in turn leads to increased insulin secretion, β-cell failure, and insulin resistance.in turn triggers hyperglycemia, which inhibits APTI activity such as hyperglycemia inhibits the activity of DHHC-7, resulting in reduced eNOS activity. **(a)** ZDHHC9 maintains the localization of GLUT1 at the plasma membrane by mediating palmitoylation of GLUT1 at the Cys207 site, thereby enhancing the cellular uptake of glucose and the rate of glycolysis. **(b)** High sugar inhibits the activity of the palmitoylating enzyme DHHC-7, leading to decreased levels of palmitoylation of endothelial-type eNOS, which in turn reduces NO secretion. This process leads to an increase in endothelin-1 (ET-1) levels, triggering endoplasmic reticulum stress and mitochondrial damage, which ultimately leads to a decrease in superoxide dismutase (SOD) activity and an increase in mitochondrial ROS levels. **(c)** CD36 possesses four palmitoylation modification sites at Cys3, Cys7, Cys464, and Cys466. During its transport from the endoplasmic reticulum to the Golgi, the newly synthesized CD36 is palmitoylated by the ZDHHC4 protein, which resides in the Golgi. Conversely, the ZDHHC5 protein on the cytoplasmic membrane hinders the depalmitoylation of CD36. **(d)** S-acylation ensures that the transmembrane sensors of Toll-like receptors are localized at the plasma membrane, and LPA activates the TLR4 receptor, which in turn activates NF-κB and promotes the expression of VCAM-1 and ICAM-1, while inhibiting eNOS activity. Additionally, palmitoylation-modified NLRP3 promotes its oligomerization, which in turn activates caspase-1 and releases IL-1β, which is involved in the inflammatory response.

Canagliflozin attenuated palmitic acid (PA)-induced vascular cellular senescence by inhibiting the activation of the ROS/ERK and iron death signaling pathways (88). In addition, it was found that ghrelin, one of the sodium-dependent glucose transporter protein 2 (SGLT2) inhibitors, was able to delay lipotoxicity-induced vascular senescence by targeting the ROS/p38/JNK pathway (89). The metabolic enzyme ethanolamine-phosphate phosphorylase (ETNPPL) was found to inhibit autophagic flux-mediated PA-induced insulin resistance in hepatocytes via the ARG2/ROS signaling cascade, suggesting that targeting ETNPPL may be a potential approach for the treatment of T2DM (90). Targeted drug therapy commonly metformin alleviates inflammation by inhibiting Fas-dependent Akt palmitoylation (91), GLP-1 receptor agonists (92, 93), SGLT2 inhibitors (94–96), and IRS-1 (97) reduce inflammation by modulating fatty acid metabolism and attenuating the negative effects of palmitic acid. PPARγ agonists (rosiglitazone), on the other hand, provide better control of glycolipid disorders by improving insulin resistance. There are also drugs that target key enzymes, such as FASN (fatty acid synthase). Orlistat enhances vascular endothelial function by reducing the intestinal absorption of palmitic acid. Meanwhile, drugs that inhibit the palmitate transporter protein (CD36) and the acylated LDL receptor (ALDLR) exert a therapeutic effect by reducing the palmitoylated modification of CD36 (98). As current pharmacological treatments have limited effectiveness in preventing limb loss, non-traditional biomarkers, including fibronectin and fatty acids, may offer insights for new therapies (**Figure 3**).

The involvement of palmitoylation modification has been well documented in experimental studies of clinical drugs. Among them, the involved palmitoylation modification sites are associated with a variety of disease organs, as shown in the table below (**Table 1**). A series of studies on key targets and pathways are important references for the development of novel drugs for the treatment of metabolic diseases and vascular injury.

**Table 1:** A collection of studies on the involvement of palmitoylation modifications in clinical drug therapy for a variety of diseases.

Drugs	Diseases	Protein acyltransferase	Palmitoylation modification site	Mechanisms
Metformin	Atherosclerosis	palmitoyl-CoA	C60	Reduction of FASN by metformin hinders Akt palmitoylation (91)
Artemether	Liver fibrosis	DHHC12	Cys18, Cys21	Induction of HSC ferroptosis via DHHC12-mediated BECN1 protein S-palmitoylation (99)
Rapamycin	Fatty liver disease	IRE1α	Cys503, Cys504	mTORC1activation triggered by protein palmitoylation (100)
Disulfiram	Myocardial infarction	ZDHHC14	Cys192, Cys191	ZHDDC14 induced palmitoylation modulated GSDMD-N-terminal cytomembrane localization (101)
Insulin	Cardiovascular disease	ubiquitin conjugating enzymes	C56S, C206S	Stimulation of palmitoylation without affecting PAFAH1b3 protein abundance (42)
Sorafenib	Liver Cancer	tyrosine kinas, ZDHHC16	Cys414, Cys600	SLC7A11, PCSK9,AKT, HippoYAP/TAZ (100)
SmStoLP-2 protein vaccine	Schistosomiasis	SmStoLP-2	Cys11, Cys61, Cys330	Enhancement of IFN-γ and TNF-α production (100)
Melatonin	Oocyte aging	almitoyl-protein thioesterase 1, APT1、APT2	Cys12, Cys354	Tubulin, miR-125a-5p/LYPLA1 (102)
Ethanol	Neuroblastoma x glioma hybrid	palmitoyl thioesterase	cys 3	Inhibition of palmitoylation of G proteins (103)
Sorafenib	Hepatocellular carcinoma	DUXAP8	Cys414	SLC7A1, p62/NRF2 (104)
Lutein	Lung tumorigenesis	DHHC20	Cys156	EGFR, PI3K, DHHC (105)
5-hydroxyfla- vone	Lung tumorigenesis	DHHC20	Cys156	EGFR, PI3K, DHHC (105)
6-hydroxyflavone	Lung tumorigenesis	DHHC20	Cys156	EGFR, PI3K, DHHC (105)

## 4 Discussion

### 4.1 Biological properties and functions of S-acylation

Protein palmitoylation, as a kind of lipid acylation modification, affects the localization, stability and function of proteins by covalently binding the unstable thioester bond of palmitic acid to specific cysteine residues of the protein substrate (4). Palmitoylation modifications are dynamically reversible, and reversible modifications are catalyzed by the DHHC acyltransferase family, which can play a key role in the dynamic regulation of protein function, localization and stability (4, 10).

Through membrane localization and signaling properties, water-soluble proteins are able to be anchored to lipid bilayers, thereby promoting the aggregation of signaling molecules within lipid rafts. Studies have shown that palmitoylated Ras proteins can activate the MAPK pathway, which in turn drives cell proliferation and differentiation (106). Characteristics of metabolic regulation include activation of fatty acid metabolizing enzymes and lipid synthases such as fatty acid synthase (107). By affecting the function of metabolism-related proteins, these regulatory mechanisms exert a modulatory effect on lipid metabolism. Palmitoylated modified SREBP-1 was found to promote the expression of cholesterol synthesis genes (108). It was also shown that DHHC4 and DHHC5 regulate fatty acid uptake and that they function in different subcellular localizations (109). The pathogenesis of metabolic diseases is usually accompanied by an inflammatory response (110). The release of inflammatory factors such as IL-6 and TNF-α can be influenced by modulating the activity of inflammatory vesicles such as NF-κB and NLRP3 (111, 112), which in turn activates caspase-1 and releases IL-1β (113). Elevated levels of free fatty acids impair insulin-mediated vasodilation and nitric oxide production (114, 115). Insulin resistance decreases arterial prostacyclin synthase and eNOS activity by increasing fatty acid oxidation in endothelial cells (116). Fatty acid synthase (FAS) levels in endothelial cells are reduced in metabolic disorders, and the absence of FAS in endothelial cells exacerbates inflammatory responses and impairs angiogenesis (117), the CD36 receptor play a key role in vascular injury (118, 119). In addition, palmitoylation modifications of metabolism-related proteins, such as glucose transporter protein 4 (GLUT4) (120) and AMP-activated protein kinase (AMPK) (121), have been demonstrated to be key metabolite markers and are important in functional studies. Excessive palmitoylation modifications may inhibit the normal function of the Akt pathway, sterol regulatory element binding protein 1c (SREBP-1c) is hyperactivated and promotes palmitic acid adulteration of triglycerides, which becomes a molecular target for lipid reprogramming in hepatocytes (122). Palmitic acid decreases peroxisome proliferator-activated receptor gamma coactivator 1α (PGC-1α) expression in blood vessels, which expression was dependent on peroxisome proliferator-activated receptor alpha (PPARα) and protein kinase A (PKA), that enhances palmitate oxidation, thereby attenuating vascular injury (123).Studies have shown that palmitic acid is able to activate pro-inflammatory pathways via membrane receptors such as Toll-like receptor 4 (TLR4) (124), a pattern recognition receptor that recognizes bacterial components including lipopolysaccharides (LPS).Palmitoylation of the TLR4 receptor enhances its localization to cell membranes, facilitates the recognition of fatty acids, and further activates the immune response that thereby triggering vascular injury (125).

### 4.2 The limitations and challenges of S-acylation

Although preclinical studies have thoroughly demonstrated that protein S-acylation significantly influences the occurrence and development of metabolic vascular damage by regulating key pathways such as the insulin signaling pathway, inflammatory response, and oxidative stress, and there is evidence that existing metabolic-related drugs (91) may partially improve vascular function by intervening in the acylation process, in-depth research in this field still faces multiple bottlenecks (126). We not only concentrate on potential therapeutic targets, such as DHHC enzymes and APT proteins, but also acknowledge that targeting palmitoylation *in vivo* for therapeutic purposes will encounter numerous challenges. These include drug specificity, off-target effects, and delivery mechanisms. Firstly, the 23 subtypes of the ZDHHC family (127), such as the DHHC4 family localized to different organelles and the deacylation enzymes APT1/2, exhibit significant spatiotemporal heterogeneity in different types of vascular cells and metabolism-related organs, their specific substrate recognition mechanisms remain unclear, and there is a lack of tissue-specific dynamic localization maps. S-acylation modifications such as acetylation, phosphorylation, and ubiquitination form a complex hierarchical network of cross-regulatory interactions, collectively influencing the activity of key targets. However, the interaction patterns of these synergistic or antagonistic effects under pathological conditions, such as in high-glucose/high-fat microenvironments have not been systematically characterized, particularly lacking a deep understanding of the competitive mechanisms at modification sites. Third, existing clinical translation models have significant limitations. Systemic ZDHHC gene knockout models struggle to accurately mimic the regional characteristics of vascular damage in human metabolic diseases, such as the differences between glomerular and retinal microvascular lesions, and cannot reproduce the dynamic evolution of S-acylation modifications during the natural progression of the disease. Furthermore, under conditions of lipotoxicity stress, the nonlinear effects of fluctuating concentrations of acyl donors, like palmitoyl-CoA, on ZDHHC enzyme activity lack corresponding quantitative models for assessment. Finally, current intervention strategies targeting acylation enzymes carry significant off-target risks. For instance, small-molecule inhibitors such as 2-bromopalmitoleic acid, which broadly inhibit the activity of multiple DHHC subtypes, may cause global disruption of intracellular signaling networks. Developing modulators with tissue-specific delivery capabilities and subtype selectivity remains a critical challenge that urgently needs to be addressed (128). As it is difficult to identify new drug targets while minimizing off-target effects, the drug development process tends to stall. The attempt to reconstruct metabolic networks is expected to provide an economical and efficient platform for testing new drug target hypotheses and effectively preventing off-target effects (129).

The mechanisms underlying the response of acylation modification to changes in the metabolic microenvironment are not well understood, particularly concerning its potential response to metabolites from the gut microbiota, such as short-chain fatty acids (130).Additionally, combining patient stratification with tracking the dynamic changes in palmitoylation may offer new therapeutic targets for personalized interventions. In the study of gut microbiota, palmitoylation acts as a key protein modification mechanism and plays a significant role. It is hypothesized that long-chain fatty acids, such as palmitic acid, can be utilized by microorganisms and converted into acetyl-CoA through the β-oxidation pathway, thereby participating in energy metabolism and synthetic metabolic processes (131, 132). However, there is currently no clear evidence indicating that short-chain fatty acids (SCFAs) in microorganisms can directly participate in palmitoylation modification, which referring to fatty acids with carbon chain lengths less than 6, such as acetate, propionate, and butyrate, are primarily produced by intestinal microbiota metabolism and play important roles in host metabolism (133). Nevertheless, research on whether SCFAs can directly participate in protein palmitoylation modification remains limited. Existing studies primarily mention palmitoylation processes involving long-chain fatty acids, such as palmitic acid and myristic acid. Regarding drug specificity, the DHHC family consists of 23 subtypes, including ZDHHC4/5/7/9/15. These subtypes display substrate preferences in vascular endothelial and smooth muscle cells. For instance, ZDHHC4 regulates STAT3 activity, and ZDHHC21 affects the palmitoylation levels of multiple enzyme systems related to vascular function. As for delivery mechanisms. Palmitation acts as a sorting signal that directs proteins to their destination, Involving metabolism, nervous system and other diseases (134–137), DHHC/APT primarily localizes to the endoplasmic reticulum-Golgi membrane system, posing a challenge for traditional small-molecule drugs to effectively reach subcellular regions. Consequently, we should develop innovative strategies, such as using lipid nanoparticles for targeted delivery of siRNA (138, 139), for example, ZDHHC5 siRNA to reduce vascular inflammation in atherosclerosis models, or employing enzyme-responsive prodrug activation systems, such as releasing APT1 inhibitors at sites of high oxLDL expression. Palmitic acid regulates cellular signaling pathways, gene expression and intracellular metabolic processes by interacting with palmitoylated modifications of proteins. The key role of gene-based regulatory mechanisms: “ZDHHC3 and ZDHHC7, localized in the Golgi apparatus, have been identified as key regulatory factors in cardiac hypertrophy because they participate in the palmitoylation process of RAC1. The enhanced activity of activated RAC1 leads to increased production of ROS, reorganizes the actin cytoskeleton, and regulates the expression of hypertrophy-related genes, thereby triggering downstream hypertrophic signal transduction during early periods of stress overload (140). ZDHHC13 has been identified as a PKM2 palmitoyltransferase, which reveals that the palmitylation process of PKM2-C31 plays a key role in PA induced endothelial injury and cardiovascular dysfunction (141). Some studies have shown that the effectiveness of literature mining methods in evaluating the proposed histoprotein-symptom matrix relationship can help predict the unexpected effects of drugs and the off-target tissues associated with their effects (142). This not only helps predict and reduce the side effects of drugs on off-target tissues, but also provides opportunities to identify new indications for target drugs.

Current research into the spatiotemporal dynamics of palmitoylation in specific diseases, such as neurodegenerative diseases and cancer, is limited. Existing literature primarily concentrates on molecular mechanisms, such as the regulation of enzyme activity, or static functional aspects, such as membrane localization. However, the relationship between spatiotemporally resolved palmitoylation regulation and disease progression necessitates further investigation. Developing detection technologies with spatiotemporal resolution capabilities, such as subcellular localization dynamic tracing techniques, will be a key component in elucidating the therapeutic window in the future.

**Figure 4:**
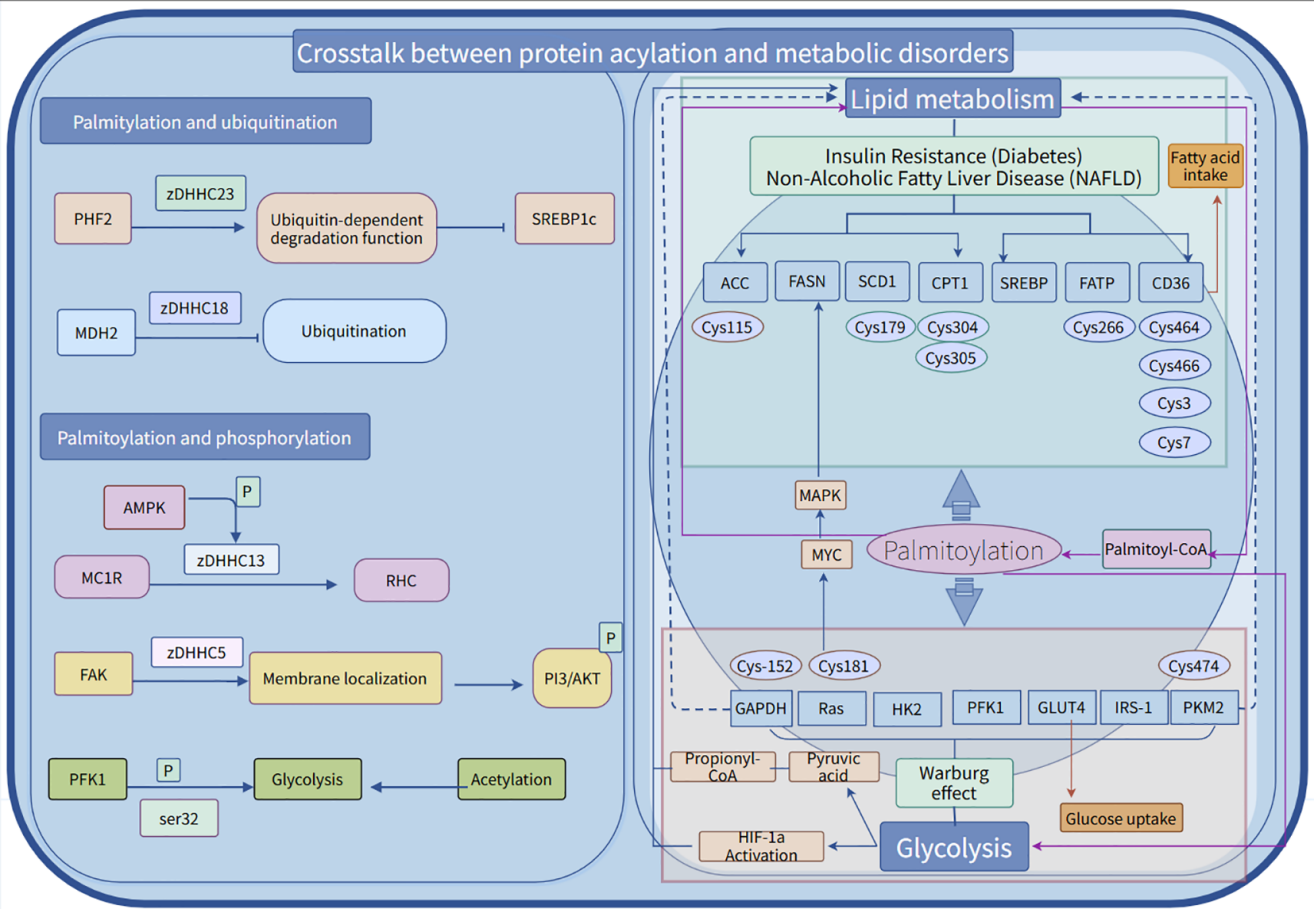
The cross-talk between palmitoylation and phosphorylation/ubiquitination and energy metabolism disorders. Palmitoylation, ubiquitination, and phosphorylation collectively uphold protein homeostasis, thereby facilitating the synergistic response of essential enzymes, such as ATP synthase, to cellular energy states. Palmitoylation plays a pivotal role in the post-translational modification of various proteins, functioning as a crucial “molecular switch” in metabolic regulation. This modification dynamically alters enzymes involved in glycolysis and lipid metabolism, modulating their activity or intracellular localization to exert specific biological effects.

Post-translational modifications (PTMs) of proteins involve the covalent attachment of functional groups to proteins, including ubiquitination, phosphorylation, glycosylation, methylation, acetylation, and glycation. These modifications affect protein stability, localization, and molecular function. Signal molecules within the cell and changes in the environment, such as phosphorylation and ubiquitination, can affect palmitylation (143). Dynamic palmitoylation indirectly affects protein stability by interfering with the ubiquitination process. Ubiquitin ligases can be modified by palmitoylation, such as E3 ubiquitin ligases PHF2 and FBXL2. When PHF2 is palmitoylated by zDHHC23, its ubiquitin-dependent degradation function is enhanced, thereby interfering with the stability of sterol regulatory element-binding protein 1c (SREBP1c) (122). Palmoylated FBXL2 was significantly enriched in the ER (endoplasmic reticulum), which promoted the degradation of IP3R3 through the ubiquitin-mediated pathway (144). zDHHC1 and zDHHC2 mediate lipid raft formation by modifying the Cys17, Cys18, and Cys246 sites of Gpm6a, thereby stabilizing the Procr protein (145); whereas zDHHC4 regulates the ubiquitinisation status of MAVS by modifying its Cys79 site, thereby enhancing stability and activating protein activity (146). The expression of malate dehydrogenase 2 (MDH2) is typically co-regulated by TRIM21-mediated ubiquitination and USP5-mediated deubiquitination. Notably, MDH2 can also be palmitoylated at the Cys138 site by zDHHC18, a modification that inhibits its ubiquitination and thereby enhances its stability (147). Palmitoylation anchors proteins to the membrane, and phosphorylation can further regulate their activity, Ras proteins require palmitoylation for localization, and then transmit signals through downstream effectors via phosphorylation.zDHHC7 catalyzes the palmitoylation of the STAT3 protein at the Cys108 residue, guiding its localization to the cell membrane rather than the nucleus. This process not only promotes the activation and phosphorylation of STAT3 but also enhances its interaction with proteins such as JAK2. In contrast, APT2 regulates phosphorylated STAT3 (p-STAT3) and facilitates its transport into the cell nucleus (148). In the crosstalk between phosphorylation and palmitoylation, G protein-coupled receptors (GPCRs) play a crucial role. Post-translational modifications of GPCRs specifically occur between phosphorylation and palmitoylation. Palmitoylation forms the fourth intracellular loop (ICL) of GPCRs through membrane insertion, a process that affects not only the receptor structure but also serves as the primary domain for phosphorylation sites. In fact, studies have shown that defects in palmitoylation significantly impair the phosphorylation process of various GPCRs (149). Palmitoylation modification at the C341 site can modulate PKA-dependent C-terminal phosphorylation and receptor responsiveness (150, 151). Similar phenomena have been reported for the 5-hydroxytryptamine (5-HT4) receptor: mutant forms that lack palmitoylation exhibit enhanced receptor phosphorylation levels both in the basal state and following norepinephrine stimulation (152). Furthermore, *in vitro* experiments have further confirmed that certain G protein-coupled receptors (GPCRs) lacking palmitoylation are more prone to phosphorylation. Studies on de-palmitoylated adrenergic receptors and rhodopsin have also found significantly elevated levels of phosphorylation in these receptors (153). Palmitoleylation is closely related to lipid metabolism and depends on palmitoleoyl-CoA, regulating ACC (acetyl-CoA carboxylase, Cys115), carnitine palmitoyltransferase 1 (Cys305), and CD36 (Cys3, Cys7), among other key lipid metabolic enzymes and signaling molecules. This affects the balance between fatty acid synthesis and oxidation, potentially leading to conditions such as insulin resistance and non-alcoholic fatty liver disease (NAFLD). Additionally, palmitoylation participates in glycolysis by modifying key enzymes or regulatory proteins, such as glyceraldehyde-3-phosphate dehydrogenase (Cys152, Cys247), pyruvate kinase 2 (Cys474), leading to metabolic reprogramming issues such as the Warburg effect, which affects cellular energy metabolism, signal transduction, and disease onset. Palmitylation serves as a cross-regulatory hub for glycolysis and lipid metabolism, A complex regulatory network is constructed between glycolysis and lipid metabolism, which affects cellular energy metabolism balance, signal transduction and disease occurrence. Its dynamic modifications are crucial in the context of diabetes, fatty liver disease, cancer, and various other conditions, making it a potential target for metabolic therapies (**Figure 4**).

### 4.3 The potential and challenges of palmitylation-related proteins as diagnostic/prognostic biomarkers for metabolic diseases

**Figure 5:**
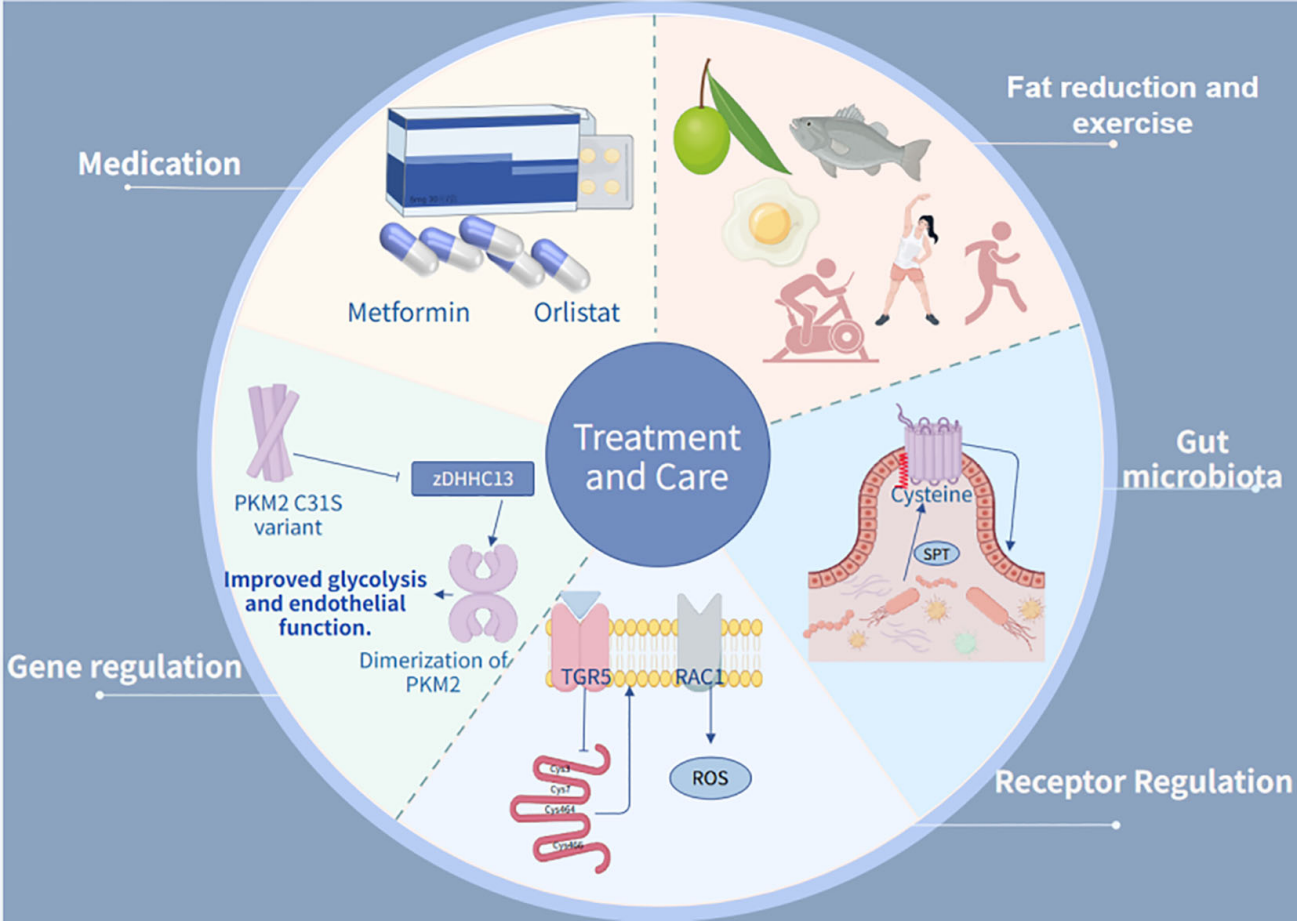
The treatment integrates therapeutic and maintenance approaches. In pharmacological management, Metformin and Insulin are employed as primary antidiabetic and lipid-lowering agents. Regarding receptor regulation, TGR5 activation is utilized to reduce fatty acid uptake. For genetic intervention, experimental studies focus on PKM2 C31S mutations. In terms of gut microbiota, serine palmitoyltransferase (SPT)-catalyzed palmitylation reactions are applied.

Dietary fatty acids and their potential to control metabolic diseases through activation of FFA4/GPR120 receptors deserve to be explored in depth. It has been shown that diabetes has a significant damaging effect on endothelial cells (154) and that interference with communication between endothelial and pericytes may lead to dysfunction of endothelial and/or pericytes. Notably, organ tissues derived from human stem cells are highly capable of restoring the structure and function of the human vasculature (155). Dietary saturated fatty acids are strongly associated with vascular damage diseases as well as type 2 diabetes, and studies replacing palmitic acid with oleic acid have shown that this replacement significantly attenuates the negative effects of saturated fatty acids on adipose tissue, skeletal muscle, liver, and beta cells (156). Results from preclinical studies suggest that dietary replacement of saturated fatty acids with a high oleic acid diet improves insulin sensitivity in humans. Combined with other lifestyle changes, this offers the possibility of reversing or delaying the deleterious effects of metabolic damage. Increased intake of olive oil, which is rich in oleic acid and contains antioxidant compounds, Therefore, dietary interventions using alternative fats, such as replacing palm oil with monounsaturated fatty acids (olive oil), may be effective in reducing fasting plasma free palmitic acid levels. In addition, time-restricted eating (10-hour restriction) may improve the efficiency of palmitic acid metabolism and reduce hepatic lipotoxicity. Focusing on dietary intake of palmitic acid and avoiding unhealthy dietary practices, such as excessive intake of saturated fatty acids, may contribute to metabolic diseases like insulin resistance and obesity by altering the gut microbiota (157) (**Figure 5**).

## 5 Conclusion

Significant advancements have been achieved in understanding the mechanisms of protein S-acylation, yet there remains ample opportunity for further studies from both basic research and clinical application perspectives. Of particular interest are the complex interactions between S-acylation and deacylating enzymes, as the normal function of most ZDHHCs and acyl thioesterases depends on a series of acylation and deacylation processes. S-acylation presents unique research opportunities for a systemic functional understanding, such as the development of novel inhibitors designed to target a specific substrate rather than modifying the enzyme (or multiple enzymes), showcasing highly promising therapeutic alternatives. the major molecular mechanisms and pathways by which palmitoylation acylation plays a regulatory role in metabolic diseases, as outlined in this review, provide a foundation for further pathological studies and the development of clinical therapeutic approaches. Future research efforts hold great potential for in-depth exploration of specific enzymes targeting palmitoylation against specific diseases. Nonetheless, there remains a lack of in-depth exploration of new methods such as metabolic reprogramming. Research on key scientific issues, including drug specificity, off-target effects, and delivery mechanisms, is insufficient to support clinical drug use requirements. Therefore, there is an urgent need to explore palmitylation-related proteins or metabolites as biomarkers for the diagnosis and prognosis assessment of metabolic diseases. Our future research will continue to monitor developments in this field and further explore this direction in subsequent studies.
