# Correction: The role of protein S-acylation in vascular injury associated with metabolic disorders

**DOI:** 10.3389/fendo.2025.1704241

**Published:** 2025-09-22

**Authors:** Yayun Wang, Wenhui Zhu, Wenfan Wang, Jiayi Zhang, Dongsen Hu, Huanmeng Shao, Yingtong Zhou, Shan Wang, Linhua Zhao

**Affiliations:** ^1^ Changchun University of Chinese Medicine, Changchun, Jilin, China; ^2^ Beijing University of Chinese Medicine, Beijing, China; ^3^ Binzhou Medical University, Yantai, Shandong, China; ^4^ National Center for Integrative Medicine, China Japan Friendship Hospital, Beijing, China; ^5^ The Affiliated Hospital to Changchun University of Chinese Medicine, Changchun, Jilin, China

**Keywords:** protein palmitoylation, palmitic acid, metabolic disorders, vascular injury, diabetes mellitus

Affiliation “Changchun University of Chinese Medicine, Changchun, Jilin, China” was omitted from the paper. This affiliation has now been added for authors “Yayun Wang^1†^, Wenhui Zhu^1†^, Wenfan Wang^1†^, Jiayi Zhang^1^”.

The original version of this article has been updated.

